# Direct Ab Initio Simulation of the Synthesis of BaZrO_3_ and the Microstructure Impacts on Proton Transport

**DOI:** 10.1021/acsnano.6c00561

**Published:** 2026-05-18

**Authors:** Rhys J. Bunting, Reetam Paul, Nikhil Rampal, Brandon C. Wood, Tadashi Ogitsu, Joel B. Varley, Tuan Anh Pham

**Affiliations:** † Quantum Simulations Group, Materials Science Division and Laboratory for Energy Applications for the Future, 4578 Lawrence Livermore National Laboratory, Livermore, California 94550, United States; ‡ Quantum Simulations Group, Physical and Life Sciences, 4578 Lawrence Livermore National Laboratory, Livermore, California 94550, United States

**Keywords:** DFT, MLIP, BaZrO3, computational synthesis, molecular dynamics, proton, transport, solid-state electrolyte

## Abstract

Controlling and predicting the processing-structure-performance relationship in functional materials is a grand challenge in materials science, with important implications for a wide range of emerging applications; a high fidelity understanding of the performance impact of microstructures formed under synthesis conditions is required to develop advanced materials, such as solid-state fuel cells and electrolyzers. Using the ceramic BaZrO_3_ as a case study, we directly simulate the synthesis and investigate how proton transport is dictated by microstructures. We develop a framework that couples density functional theory (DFT), machine-learning interatomic potential (MLIP) driven molecular dynamics, and grand canonical Monte Carlo to perform large-scale, microstructure-resolved, atomistic simulations of proton transport in experimentally representative polycrystalline structures. Our fully *ab initio* approach, using a MLIP as a proxy for DFT, allows us to quantify the competition between two distinct diffusion mechanisms: one associated with grain-boundary regions and another within grains. When the impacts of grain boundaries are taken into account, proton transport exhibits substantial deviation from the bulk oxide limit. This addresses long-standing discrepancies between theory and experiments. Our integrated approach provides atomistic insight into microstructure-dependent proton pathways in BaZrO_3_ and establishes a general protocol for predicting processing-structure-performance relationships.

## Graphical abstract

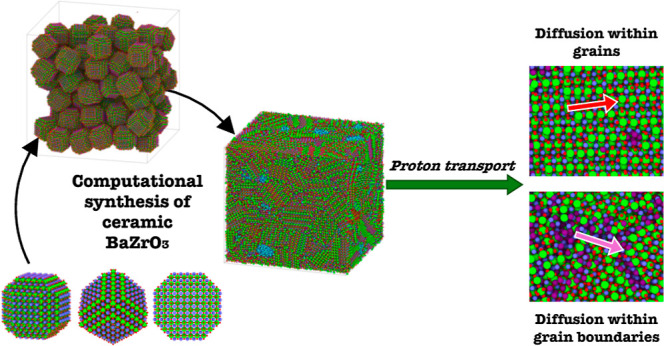

## Introduction

Hydrogen fuel cells and water electrolyzers are capable of transforming energy security.^1–8^ They accomplish this through increased feedstock availability, high energy density, and exceptional energy conversion efficiency. For both of these processes, there must be a nonconductive separation of the anode and cathode to prevent short circuiting. To complete the reaction, protons must move from the anode to the cathode. An optimal material should conduct protons efficiently while remaining an electrical insulator.

One class of materials used for this is proton-exchange membranes.^9^ These membranes are often polymers that are engineered for properties such as stability/chemical inertness, flux of proton transport, low cost, and electrical conductivity. While these membranes have had much success, they still are limited by their stability under high temperatures and redox potentials. One avenue for overcoming these issues is to use a solid state proton-exchange membrane made with inorganic materials.^10^

Barium zirconate (BaZrO_3_) is a promising proton-conducting ceramic electrolyte for fuel cells.^11–13^ It combines high-temperature stability with appreciable proton mobility, although further improvements are needed to boost performance and mitigate transport-limited losses. To this end, defect-engineering strategies, such as introduction of controlled vacancies and acceptor dopants, are used to optimize materials structures and facilitate proton migration. Dopants, predominantly yttrium, are used to induce oxygen defects in the lattice, with oxygen vacancies assumed to be equal to the amount of dopant added (often up to 0.2 molar).^14^ Extensive experimental and theoretical studies have probed the mechanism of proton diffusion in these systems.^14–21^ However, discrepancies remain for experimentally measured activation energies and theoretical predictions. Values of 0.44 eV^15^ and 0.47 eV^16^ are found experimentally, while for theory, a wide range of barriers of 0.60 eV,^17^ 0.22 eV,^18^ 0.83 eV,^19^ 0.25 eV,^20^ and 0.45 eV are found.^21^ Resolving this gap is essential for the rational design of high-performance ceramic proton conductors.

Theoretical models based on first-principles typically employ density functional theory (DFT), offering a balance between computational cost and accuracy.^22^ However, with the cubic scaling of DFT calculations, the system size is typically limited to the order of hundreds of atoms or less. Accordingly, simulation models often adopt small supercells of the crystal unit cell, which, while quite representative of single crystals, are inherently more idealized than real systems exhibiting compositional and structural diversity. While progress has been made in understanding nonidealities, such as the impacts of isolated defects, dopants, and defect complexes, *ab initio* models still provide simplified descriptions of most experimentally synthesized materials, particularly where complex microstructure and grain boundaries are present due to the synthesis methods. For example, synthesis efforts utilizing powders and other noncrystalline precursors often provide cost-effective routes for engineering materials with desired morphologies, porosities, and densities, which inevitably leads to significant populations of grain boundaries and microstructures.^23^ These types of materials have remained largely challenging to model in representative systems due to challenges in extending DFT-based simulations to relevant time and length scales.

Other theoretical models try to overcome these limitations. For atomistic simulations, classical potentials are used, where atomic interactions are described by well-defined functions which are often fitted to experimental or theoretical data.^21^ Both fitting efforts come with additional complications to describe the proper physics in the constrained functional form that may not be adequately representative of the potential energy surface. In addition, with the high dimensionality of grain boundaries, it is exceedingly difficult to describe a polycrystalline system, where many distinct grain boundaries will be present.^23^ Beyond atomistic simulations, transport properties can be described within mesoscale or continuum level models,^24^ where microstructure effects can be interrogated but this requires parametrization, often via atomistic simulations. This indicates a need for new methodologies that can consider the transport properties of structurally complex materials across scales that directly incorporates the role of microstructure.

To this end, we describe an approach utilizing DFT, machine learning interatomic potentials (MLIPs), and grand canonical Monte Carlo (GCMC) to simulate the structure-dependent transport of protons in BaZrO_3_ with atomistic-level precision and insight. Our approach includes building a data set using DFT to describe polycrystalline and defected BaZrO_3_, which is used to train a MLIP to rapidly simulate across far larger length- and time-scales than those accessible with DFT. With these potentials and molecular dynamics coupled with GCMC, we initially simulate the synthesis of a sample by heating and compressing monodisperse nanoparticles of BaZrO_3_ as a precursor. We also evaluate proton diffusion using molecular dynamics for pristine and oxygen-deficient single crystal BaZrO_3_ and compare this to the *in silico* synthesized pellet, where we use site classification within the samples to correlate the transport with local crystallinity. We show explicitly that representing grains and grain boundaries in the simulations is essential to achieve quantitative agreement with experiment. Additionally, the calculations reveal two distinct regimes: proton motion confined to grain-boundary networks; and intragranular (bulk) diffusion.

## Results/Discussion

A diverse variety of structures in the data set (Figure 1a) allows our MLIP to properly describe polycrystalline BaZrO_3_ (Figure 1b). The trained potential has a test set mean absolute error of 0.146 eV Å^–1^ for forces and 31.34 meV per atom for energy (further training analysis can be found in the Supporting Information S1/2). Additionally, the potential was validated with ground truth AIMD simulations of a proton in a smaller 2 × 2 × 2 supercell of pristine BaZrO_3_. Shown in Figure 1c, a good agreement in the radial distribution functions (RDFs) of the elementary species between MLIP and AIMD simulations indicates that the generated potential can effectively describe proton structure in BaZrO_3_. The potential is also verified against calculations of the (100), (110), and (111) surfaces and the stacked surfaces to form respective grain boundaries (Supporting Information S2). These pristine surfaces and grain boundaries are out of data set for the potential, yet the potential correctly orders the stability of these. However, the overall trend is that the potential predicts surfaces to be more stable and grain boundaries to be less stable.

**Figure 1:**
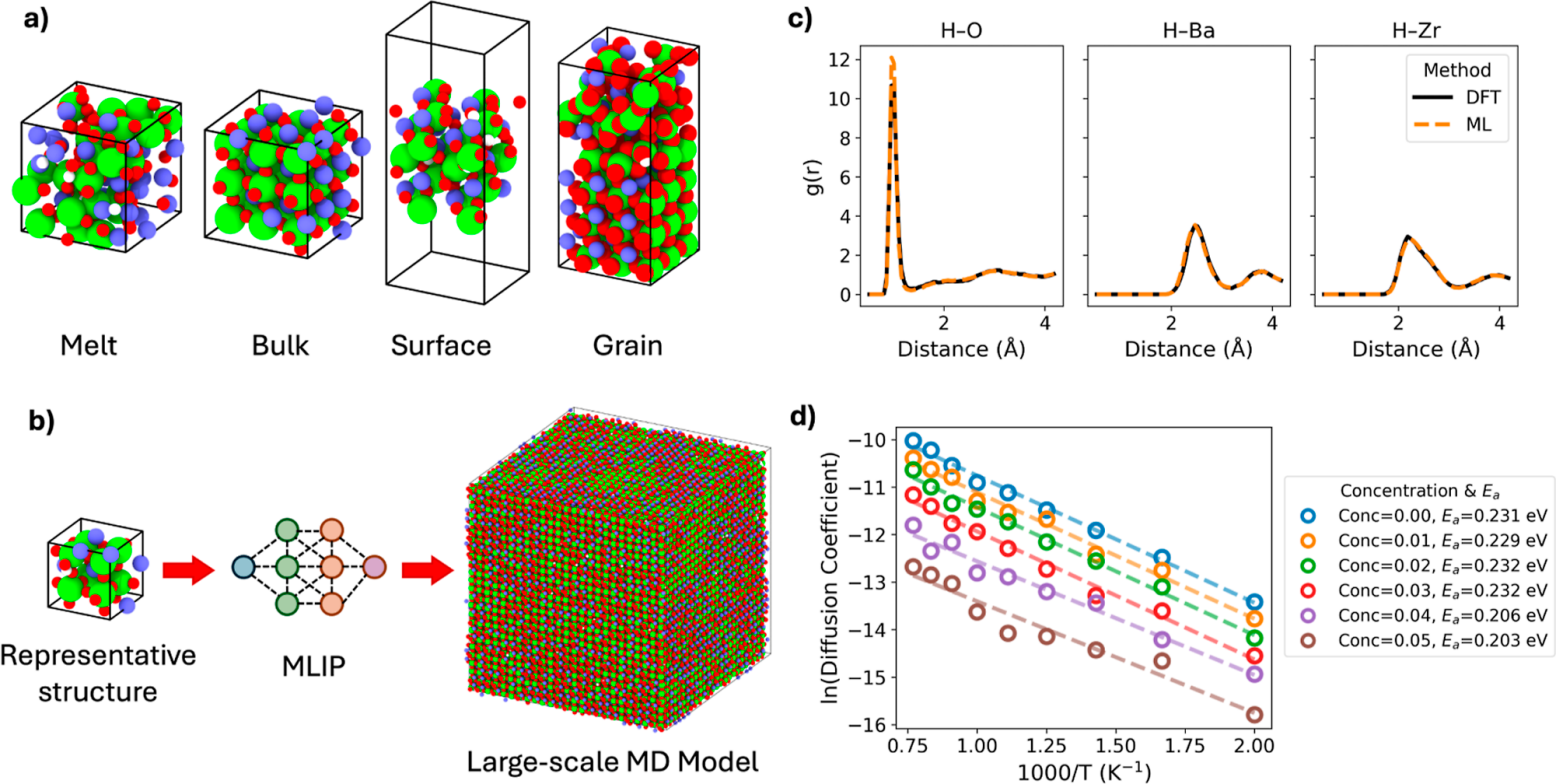
(a) Example of atomic structures used in the DFT based data set. (b) Simulation sizes used at the DFT level and ML level of theory. (c) Validation of the ML potential against the DFT ground truth for a proton in a 2 × 2 × 2 supercell shown in (b). (d) Arrhenius plot of the diffusion coefficient of protons in the bulk structure shown in (b), with varying oxygen vacancy molar concentrations considered.

A key metric for evaluating the proton exchange capability of a solid-state electrolyte is the activation energy for proton diffusion. This quantity can be determined from the Arrhenius relationship between the diffusion coefficient and temperature. The diffusion coefficient was obtained from the mean squared displacement (MSD) of protons, calculated from MD trajectories. Focusing first on pristine bulk BaZrO_3_, the trained MLIP was employed to simulate proton diffusion in the system modeled using a 25 × 25 × 25 supercell containing 0.05 molar fraction of hydrogen (a total of 78,906 atoms). Simulations were performed across experimentally relevant temperatures ranging from 500 to 1300 K, each with a minimum sampling duration of 5 ns to ensure statistical convergence. Shown in Figure 1d, the proton diffusion coefficient in pristine BaZrO_3_ increases monotonically with temperature across the investigated temperature range, consistent with Arrhenius-type behavior. From the slope of this plot, the activation energy for proton diffusion in pristine BaZrO_3_ was determined to be 0.23 eV. This value falls within the broad range of activation energies reported in previous theoretical studies employing various computational methods.^18–21^ However, it is notably lower than the experimental activation barrier of 0.44 eV reported for fitted undoped BaZrO_3_.^14^ Our result is closer to an experimentally measured activation barrier of 0.16 eV for yttrium-doped BaZrO_3_ that better approximates a proton-trap-free environment.

To reconcile the discrepancy between the calculated and experimentally measured activation energies for proton diffusion in pristine BaZrO_3_, the role of oxygen vacancies is examined. It is well established that oxygen vacancies (*V*_O_) strongly influence the proton transport properties of BaZrO_3_ and that defect engineering via yttrium doping is commonly employed to enhance the concentration of *V*_O_ and proton uptake.^25,26^ However, the increased population of defects can also lead to more proton trapping sites that can impact the energy barriers.^27^ This is expected to be dominated by *V*_O_ (and *Y*_Zr_ acceptors) considering the much lower concentrations expected for barium and zirconium vacancies.^25,26^ Here, to investigate impacts of oxygen defects on proton transport, we randomly generated oxygen defects throughout the aforementioned supercell structure, with *V*_O_ concentrations considered up to 0.05 molar fraction, where a lower concentration is used to minimize spurious interactions. These results are presented in Figure 1d, where the proton diffusion coefficient decreases from 4.42 × 10^–5^ cm^2^ s^–1^ to 3.12 × 10^–6^ cm^2^ s^–1^ for increasing *V*_O_ concentrations at 1300 K. As expected, the trend sees diffusion being negatively correlated to *V*_O_ populations, with their presence slowing down the rate of diffusion. The diffusion for these systems still exhibits Arrhenius behavior, with fitted activation energies staying within the range of ∼0.2 eV, with a slight decrease from 0.23 to 0.20 eV from the pristine to the 0.05 molar *V*_O_ within a consistent sampling time. These results suggest *V*_O_ populations cannot account for the deviations between the experimental and theoretical results, although we note we are not explicitly accounting for yttrium-doping in our model.^14,16^

One possible cause of this discrepancy is that the simulation model does not properly represent the complexity of realistic systems. In particular, synthesized materials have point defects, stacking faults, and grain boundaries which are not included in typically used models.^28^ To overcome this limitation, a model of BaZrO_3_ pellet formation with experimentally relevant inputs is considered.^29,30^ A pseudopowder is made from monodisperse nanoparticles of BaZrO_3_ (sized 4.2 nm),^31^ which are modeled through Wulff construction of the most stable surfaces.^32^ The pseudopowder model consists of 100 randomly packed BaZrO_3_ nanoparticles, comprising a total of 396,300 atoms. The system was subjected to MD simulations under experimentally relevant pellet-pressing conditions, corresponding to a pressure of 10,000 bar and a temperature of 2000 K.^33–35^ The simulation was performed for a minimum duration of 4 ns to ensure structural equilibration.

Shown in Figure 2a,b, most of the pellet formation process happens within the first 20 ps, where mechanical movement of the nanoparticles coming more closely together drives pellet formation from the pseudopowder. This can be observed with the dramatic fluctuations of the pressure applied by the barostat during this time, where movement of the nanoparticles into cavities releases stress from the system (Figure 2a). The process of the pellet formation was completed after ∼100 ps of simulation time. This is affirmed by two pieces of data: the sample density plateauing at ∼5 g cm^–3^, with the density effectively doubling from its initial density (Figure 2b); and the standard deviation of enthalpy also plateauing (Supporting Information S3). A very low powder density is observed at the start of the simulation, based on the poor initial packing. An initial higher density may have been obtained if there was more stringent optimization of the packing, such as running MD simulations under standard pressure. The density quickly increases to ∼5.0 g cm^–3^ by 100 ps (see Figure 2b), only changing very slightly beyond this point, indicating that the densification process is effectively complete. Densification with respect to the experimental single crystal density reaches 88.5%, in good agreement with the representative experimental values (e.g., 92% in ref 16).

**Figure 2:**
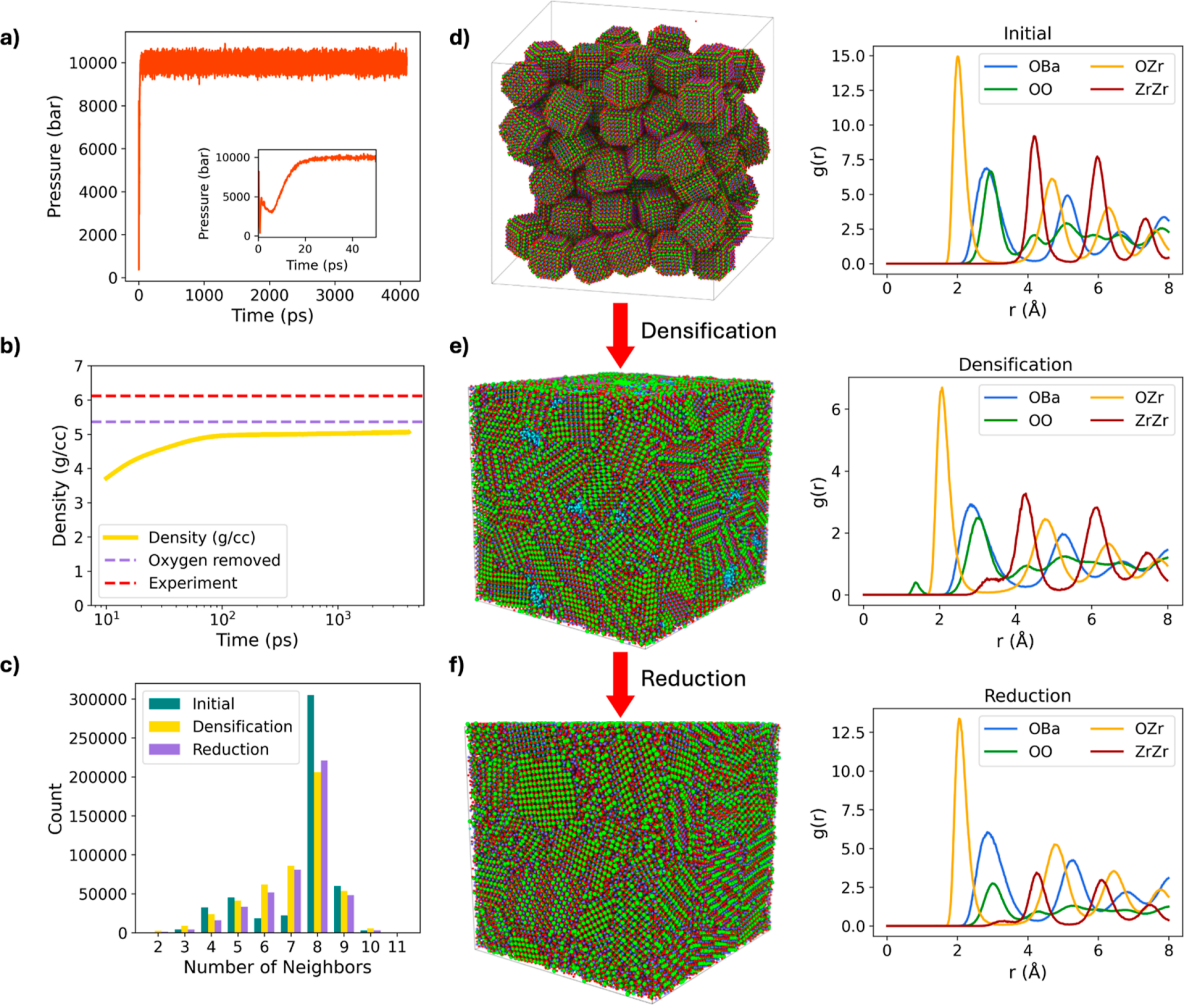
(a) Pressure applied with the barostat during the densification simulation. (b) Density of the system during the densification simulation, along with the density post reduction and the calculated experimental single crystal density. (c) Nearest neighbor coordination number for oxygen with neighboring oxygen atoms correlating to the first minima of the radial distribution function (d–f) for the initial powder, compressed pellet, and the oxygen reduced pellet. (d–f) Structure of the initial powder, compressed pellet, and the oxygen reduced pellet, respectively, along with their corresponding radial distribution functions.

While the change in density can map the mechanical processes that took place, the chemical processes must also be investigated. To do this, nearest neighbor coordination number analysis is performed for oxygen. In the initial powder state sampled for the first 10 ps, there is a bimodal distribution, with peaks at 5 and 8 nearest neighbors (Figure 2c). The peak at 8 neighbors correlates to bulk lattice oxygen; the oxygen atoms that are in the bulk of the nanoparticles. The other atoms correspond to surface/subsurface sites that are under-coordinated. Interestingly, in the final structure after densification, sampled for the final 100 ps, unimodal distribution occurs. This is due to the removal of surface sites, with all oxygen atoms being present within the condensed phase. The distribution is also less sharp, which is caused by the distorted and polycrystalline nature of the final structure upon densification.

RDFs of all species are also calculated for the initial pseudopowder and the formed pellet (Figure 2d,e). All peaks in the RDFs maintain their location in both the initial and final structures. There is a flattening of the peaks, with this being explained by strain and distortions formed in the polycrystalline structure. This strain would be especially prominent along grain boundaries. Notably, two new peaks are found in the RDFs after compression, evidence of new structural chemistry compared to the pristine nanoparticle initial state. In the Zr–Zr RDF, a small, broad peak emerges at approximately 3.3 Å. This feature is attributed to a stacking fault arising from the termination of the (100) facets of the nanoparticles-used in the pseudopowder model; they terminate on the Zr atomic layer. When two such (100) facets come into contact during the simulation, their interaction leads to the formation of this stacking fault, which manifests as the additional peak in the RDF.

Additionally, in the O–O RDF, a new peak appears at approximately 1.3 Å, corresponding to the formation of molecular oxygen (O_2_). The most stable termination of the BaZrO_3_ surface is oxygen-terminated, resulting in an excess of oxygen atoms within the system. Under compression, these oxygen-terminated surfaces come into contact, promoting oxygen recombination and the formation of O_2_ species. During the simulation, the generated molecular oxygen accumulates within intergranular pockets along the grain boundaries, as illustrated by the cyan-highlighted O_2_ molecules in Figure 2e. Although O_2_ pockets are observed in the simulation, this behavior is nonphysical under experimental conditions. In practice, any gases formed during the pressing process would escape from the sample, whereas in the simulation, the use of periodic boundary conditions creates a closed system that prevents gas release. To more accurately represent the experimental environment, the grand canonical ensemble (GCMC–MD) was employed to maintain a constant oxygen chemical potential (see Supporting Information). The oxygen potential was set to −8.047 eV, corresponding to conditions typical of low-oxygen/argon-rich annealing environments. The simulation was continued until the oxygen mass reached a steady state, which occurred within approximately 8 ns.

Before oxygen removal, the pseudopellet has 18,127 close contacted oxygen atoms. Note that some of these atoms correspond to surface oxygen species with adsorbed molecular oxygen nearby; not all close contacts represent true molecular O_2_. After oxygen removal, the number of closely contacted oxygen pairs decreased to 210, resulting in an overall stoichiometry of Ba_1.04_Zr_0.96_O_3.03_. The remaining molecular oxygen species, colored cyan in Figure 2f, are sparsely distributed throughout the structure and therefore not readily visible. These oxygen species are primarily located along grain boundaries and are absent within the bulk grain interiors. The removal of molecular oxygen is also reflected in the RDF, where the O–O peak near 1.3 Å disappears and the remaining oxygen-related peaks become sharper (Figure 2f). A similar effect is observed in the nearest-neighbor coordination analysis, where the peak at 8-fold coordination becomes more pronounced (Figure 2c). With this oxygen removal, annealing of the pseudopellet is complete and ready for consideration of its proton transport properties.

With the polycrystalline nature of the pseudopellet, there are crystalline bulk-like and noncrystalline grain boundary regions between the grains formed from the initial nanoparticle mixture. These regions must be classified to give insight into the diffusion properties of the sample. To allow classification of the local atomic environments, SOAP descriptors are generated for all oxygen atoms, with a cutoff radius of 3.6 Å chosen. These descriptors are made for simulation snapshots of a pristine structure of BaZrO_3_ (Figure 1b) and for the structure of the pseudopellet after oxygen removal (Figures 2f and S4).

UMAP dimensionality reduction was applied to the generated SOAP descriptors (Figure 3a). Visual inspection of the resulting manifold reveals the presence of two distinct clusters. To quantitatively confirm this observation, DBSCAN was performed on the UMAP-reduced data, which likewise identified two primary clusters. These clusters correspond to two distinct local structural environments: an ordered crystalline phase and a strained, disordered phase. A small fraction of oxygen atoms from the nominally crystalline data set appear within the disordered cluster (see Supporting Information S4). This is attributed to transient events in which oxygen atoms come into close proximity with one another under the high simulation temperature of 2000 K, leading to brief local distortions. The oxygen atoms classified as part of the disordered phase are highlighted in purple in the atomic configuration shown in Figure 3b. These disordered atoms are predominantly located at grain boundaries, consistent with their identification as intergranular, noncrystalline regions. Further quantification of the grain boundaries is available in Supporting Information S5.

**Figure 3:**
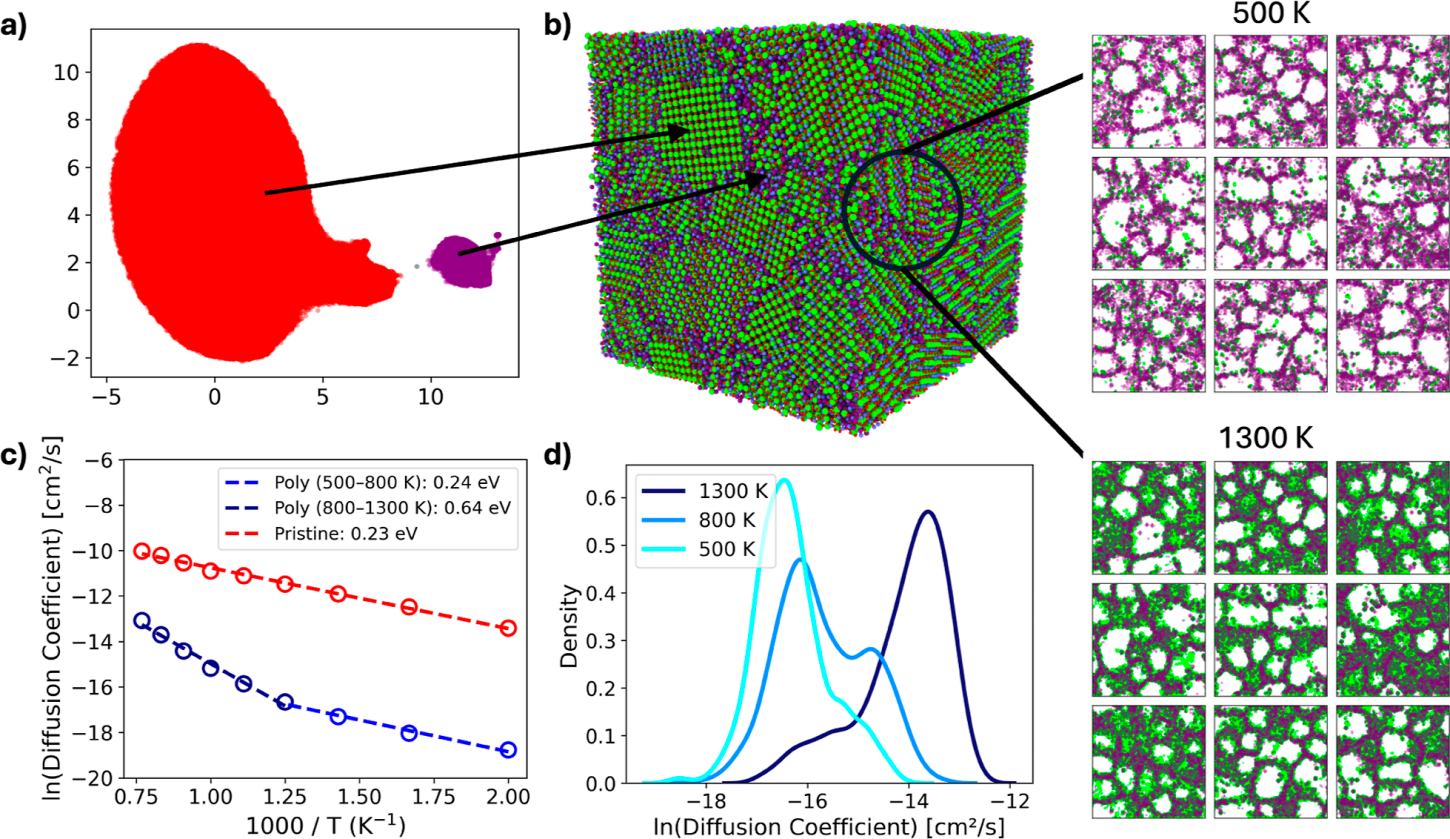
(a) UMAP and subsequent DBSCAN clustering of SOAP descriptors of oxygen atoms in the computationally synthesized pellet. Crystalline oxygen atoms are classified as red and noncrystalline atoms are classified as purple. (b) Structure of the computationally synthesized pellet (left), with noncrystalline/grain boundary oxygen atoms colored purple. Sliced structure of the computationally synthesized pellet. Noncrystalline/grain boundary oxygen atoms are marked in purple, with proton concentration during diffusion marked in green for 500 and 1300 K. (c) Arrhenius plot of the diffusion coefficient of protons in the bulk structure and polycrystalline structure. Two fits are made for the polycrystalline structure due to the non-Arrhenius nature. (d) Diffusion modes of protons when the diffusion of each proton is considered.

Proton diffusion is then considered for the pseudopellet. Shown in Figure 3c, the diffusion coefficient is ∼20 times lower for the polycrystalline BaZrO_3_ at 1300 K, corroborating to previous results that suggest that microstructure is a crucial part of the material’s effectiveness.^21^ Diffusion coefficients for models of the idealized grain boundaries can be found for reference in Supporting Information S5.4. Interestingly, upon consideration of the Arrhenius plot, the polycrystalline structure shows a more complex behavior that indicates a mechanistic change occurring around 800 K, with two different slopes for temperatures above and below ∼800 K. To better understand the mechanism of proton diffusion, a heatmap of proton density in the polycrystalline structure is performed at 500 and 1300 K. Shown in Figure 3b, a heatmap is presented as slices along the *y* axis, with the proton heatmap marked in green and the noncrystalline oxygen atoms/grain boundaries marked in purple. Proton diffusion is apparently slowest at the grain boundaries and is the reason for the higher proton concentration. This slower proton diffusion can be explained by the presence of under-coordinated oxygen atoms present along the grain boundaries. Adsorbates typically bind more strongly to under-coordinated species, explaining the slower proton transport in these regions.^36^ At higher temperatures, more diffusion takes place, causing larger smearing of proton density in the structure.

Neglecting the transition between these transport modes, the overall fit for the activation energy is found to be 0.40 eV, in good agreement with the experimentally reported value of 0.44 eV.^14^ However, we stress that our simulations offer a more complete understanding of proton diffusion in a polycrystalline sample for different temperatures. Specifically, the results suggest two diffusion modes are present, with the dominant diffusion mode changing with temperature, with 800 K as the inflection point. From the Arrhenius plot in Figure 3c, these two mechanisms have effective barriers of 0.23 and 0.64 eV, respectively, for the low and high temperature regime, explaining the two apparent activation processes.

One activation energy correlates to the barrier required to diffuse through the grain boundaries in the polycrystalline sample. The other activation energy correlates to bulk diffusion within the grains. At high temperatures, there is enough thermal energy for protons to pass through the grain boundary in the simulation time scale. However, at lower temperatures, this process becomes a rare event as there is not enough thermal energy, causing protons to simply rattle within the grains. Experimentally, when the activation energy of grain boundaries and bulk conduction are separated, values of 0.65 and 0.43 eV are found, respectively, for yttrium-doped BaZrO_3_.^37^ An effective barrier, combining both, finds a value of 0.68 eV. They also find a point where there is an inflection where grain or bulk diffusion is the limiting factor, at ∼500 K. These experimental findings closely corroborate our findings in this work; an effective barrier of 0.64 eV versus the experimental 0.68 eV is found. While we also find an inflection for the limiting diffusion step, it is ∼300 K higher than the experimental one. The difference found in the bulk diffusion activation energy, of 0.43 eV vs 0.23 eV could be explained by the proton-trapping. Other experimental work, that accounted for proton-trapping caused by yttrium doping found an effective barrier of 0.16 eV, closely corroborating with our bulk diffusion value.^16^ In our simulations, diffusion, and not conductivity, is considered. With this, there is no flow of protons where passing through grain boundaries is necessary; the protons can remain and move within the bulk regions in the grains. Due to this, when passing through the grain boundaries becomes prohibitive at lower temperatures, only diffusion within the bulk region in the grains takes place. This causes an activation energy for only the bulk region to be calculated, and not the effective barrier for both the grain and grain boundary diffusion. In this region, diffusion is limited by transport through the grains.

Finally, it is important to note that additional effects might impact the predicted activation energy. More stable grains may dominate under the long time scales of thermal annealing, where these more stable grains may have lower apparent activation energies for diffusion. Experimental grains are also much larger (micron/submicron size), changing a proton’s time within the grain boundary and bulk diffusion modes, which would change the effective barrier.^23^ To a much smaller effect, the discrepancy could involve some method limitations, such as the DFT functional used;^38^ machine learning potential error; and lack of nuclear quantum effects.^39^ Nevertheless, we note that the observed activation energies for bulk proton transport, as parametrized here via the PBE generalized gradient approximation (GGA) exchange correlation functional, are in excellent agreement with previously reported values calculated with hybrid functionals, suggesting a small impact of the exchange–correlation functional in DFT simulations.^27^

## Conclusions

We develop and propose an approach using a suite of computational tools to attempt to more faithfully connect material structures formed during synthesis with experimental observables. Using proton transport in the ceramic BaZrO_3_ as an example, our results reinforce how crucial it can be to consider the microstructure of materials to describe the macroscopic properties, particularly for systems beyond single crystals where structural complexity can dominate the observed performance. In this work, we go beyond the standard assumption of a given structure by also simulating the synthesis of the material; this allows some conciliation between experimental and theoretical work, showing different mechanistic transport regimes due to structural features that can account for experimental observations. With machine learning enabling the scalability of first-principles electronic structure methods, we hope that more emphasis is placed on building more representative atomistic models of the physical system instead of simply accelerating simulations.

For BaZrO_3_, the results shed light on the well-known role that microstructure plays in impacting proton conductivity and the importance of microstructure engineering to enhancing performance. While single crystal synthesis remains outside industrial applications, our results suggest the benefit of increasing the grain sizes of BaZrO_3_ in solid state proton membrane applications. There may be other materials available that are inherently worse at proton transport in their pristine form, but their grain boundaries have less of an effect on ion transport properties. This work also raises questions on the effect of dopants along grain boundaries for diffusion properties.

Beyond proton-exchange membranes, we envisage the implementation of MLIPs to consider more physically realistic models that are faithful to specific experimental conditions. There is now an intersection between experimental and theoretical work: experimental systems are getting smaller at the nanoscale; ab initio theoretical methods are now capable of considering models beyond this scale, providing atomic-level insight into macroscopic behavior. There are opportunities to consider other systems using methods we employed, such as in the field of batteries,^40^ catalysis,^41^ material physical properties,^42^ and self-assembly.^43^

## Methods/Experimental

### Ab Initio Static & Molecular Dynamics Calculations

Density functional theory (DFT) calculations were carried out using the Vienna ab initio Simulation Package (VASP)^44–46^ with the Perdew–Burke–Ernzerhof (PBE)^47^ exchange–correlation functional within the generalized gradient approximation (GGA). The projector augmented-wave (PAW) method^48,49^ was employed to describe the interaction between core and valence electrons. A plane-wave cutoff energy of 400 eV was used for all calculations. The Brillouin zone was sampled using the Monkhorst–Pack scheme^50^ with a 3 × 3 × 3 *k*-point mesh for bulk and melt structures, and a 3 × 3 × 1 mesh for all surface and interface models. All ab initio molecular dynamics (AIMD) simulations were sampled at the gamma point. Fermi–Dirac smearing was applied for AIMD simulations, whereas Gaussian smearing was used for static calculations. AIMD simulations were performed in the *NVT* ensemble using a Nosé–Hoover thermostat to maintain temperature control.^51^

### Machine Learning Potential Development

An initial data set was constructed from optimized DFT structures of bulk and low-index surfaces, including the (100), (110), and (111) facets. Here, the bulk configurations were modeled as 3 × 3 × 3 supercells, while the surface slabs were built as *p*(3 × 3) for (100), *p*(3 × 2) for (110), and *p*(2 × 1) orthogonal supercells for (111), each separated by at least 12 Å of vacuum. For each configuration, 1000 structures were generated by successively (i) applying Gaussian perturbations to atomic positions, (ii) introducing point defects through atom deletions, and (iii) performing proton insertions. An initial MLIP was then trained on this data set and subsequently used to generate additional configurations. Specifically, 100 representative structures were selected for molecular dynamics (MD) simulations conducted at 2500 K with a 0.25 fs time step for 12.5 ps. Ten snapshots from each trajectory were extracted, yielding an additional 4000 configurations.

To further expand the data set, 100 melt-phase structures of BaZrO_3_ were generated using AIMD at 10,000 K with a 0.20 fs time step over 10 ps. Additional melt configurations were obtained by applying the same structural generation protocol used for the initial data set. A second MLIP was then trained on all accumulated data and used to generate new structures for bulk, surface ((100), (110), (111)), and interfacial systems encompassing all possible facet and termination combinations. The same data generation procedure was followed, but at 700 and 2500 K, with interfaces additionally subjected to 10,000 bar of compression along the *z*-axis using a Nosé–Hoover barostat. In addition, 100 molten structures of elemental Ba and Zr were sampled at 3500 and 2500 K, respectively, using the second MLIP with a 1 fs time step for 100 ps. Likewise, 100 configurations of elemental oxygen and hydrogen were generated via AIMD simulations at 2500 K with a 0.5 fs time step for 5 ps, containing 16 gas molecules in each cell. A final MLIP was trained on the combined data set after filtering out samples with atomic forces exceeding 50 eV Å^–1^. The resulting data set comprised 23,404 structures, partitioned into 85% for training, 10% for validation, and 5% for testing. This final potential was employed for all subsequent simulations and analyses.

All MLIPs were trained using the Allegro package^52^ and implemented within LAMMPS.^53^ Allegro version 0.5.0 was used, coupled to torch version 2.7.0. Training was finished after 233 epochs. Atomic environments were represented with a cutoff radius of 5.5 °A, employing eight Bessel functions with a polynomial envelope function of *p* = 5. A total of 64 scalar and 32 tensor features of even parity were included, with the maximum rotational order truncated at *l*_max_ = 2. The network architecture comprised one interaction layer. The two-body latent MLIP utilized two hidden layers with 64 nodes each, while the latent network head consisted of four output layers, each containing 32 nodes. Training was performed with a batch size of 1 and an initial learning rate of 0.001. Additionally, the loss function assigned a weighting ratio of 10:1 between force components and total energy terms to prioritize accurate force reproduction during training. A hexbin plot of the potential error can be found in Supporting Information S1.

### Polycrystalline Formation and Proton Transport Simulations

Proton transport in both pristine and defective BaZrO_3_ was simulated using a 10 × 10 × 10 supercell. All MD simulations employed a time step of 0.5 fs and a total simulation time of 5 ns for pristine and 1 ns for polycrystalline. For polycrystalline BaZrO_3_, the initial structure was constructed by packing 100 BaZrO_3_ nanoparticles using the Packmol package. Each nanoparticle was based on the experimentally validated Wulff construction, corresponding to the most stable morphology for a particle diameter of approximately 4.2 nm.^32,54^ The system was equilibrated at a temperature of 2000 K and a pressure of 10,000 bar, with a time step of 0.5 fs. This is followed by grand canonical Monte Carlo–molecular dynamics (GCMC–MD) simulations, where five exchange attempts were sampled at each MD step under identical thermodynamic conditions (2000 K and 10,000 bar).^55^ The grand canonical approach is implemented to simulate the reduction of the pellet under an argon environment, where excess oxygen is removed. The oxygen bath chemical potential was defined relative to oxygen behaving as a harmonic free gas with a concentration equivalent to 1 ppm, yielding an effective potential of −8.047 eV. Oxygen removal from the simulation plateaued after approximately 8 ns, at which point the run was terminated. Finally, proton transport was simulated using the same protocol as the pristine system.

To analyze the resulting atomic environments of the polycrystalline systems, Smooth Overlap of Atomic Positions (SOAP) descriptors were computed with the DSCRIBE package.^56,57^ A cutoff radius of 3.6 Å was applied, with six radial basis functions and a maximum angular momentum quantum number l = 4. The sigma for the Gaussian smearing is set to 0.5. The Uniform Manifold Approximation and Projection (UMAP) method^58^ was then used to reduce the SOAP feature space to two components, with 100 nearest neighbors and a minimum distance parameter of 0.1. For unsupervised structural classification, Density-Based Spatial Clustering of Applications with Noise (DBSCAN) was performed using the scikit-learn library.^59,60^ A maximum neighborhood distance (σ) of 0.5 was used, and a minimum cluster size of 100 samples was required for assignment as a cluster; smaller populations were designated as noise.

## Supplementary Material



## Notes

Machine learning interatomic potential training metrics with benchmarking of surface and grain boundary energies to the ground truth. Enthalpy convergence of densification simulation. UMAP of SOAP descriptors of the crystalline and polycrystalline system. Further grain boundary analysis (PDF)
