# Supplementary material for: Direct Ab Initio Simulation of the Synthesis of BaZrO3 and the Microstructure Impacts on Proton Transport
Source: ACS Nano. 2026 May 18;20(21):15181–9. doi: 10.1021/acsnano.6c00561 (PMC13235642; doi:10.1021/acsnano.6c00561)
Supplement: Supplementary file 1 [file nn6c00561_si_001.pdf]

Supplementary Information – Direct Ab Initio Simulation of the Synthesis of $BaZrO_3$ and the Microstructure Impacts on Proton Transport

Rhys J. Bunting,[*,†] Reetam Paul,[‡] Nikhil Rampal,[†] Brandon C. Wood,[†] Tadashi Ogitsu,[†] Joel B. Varley,[†] and Tuan Anh Pham[*,†]

†*Quantum Simulations Group, Materials Science Division and Laboratory for Energy Applications for the Future, Lawrence Livermore National Laboratory, Livermore, California 94550, United States*

‡*Quantum Simulations Group, Physical and Life Sciences, Lawrence Livermore National Laboratory, Livermore, California 94550, United States*

E-mail: bunting4@llnl.gov; pham16@llnl.gov

Contents

Machine learning interatomic potential training **3**

Surface and grain boundary energies **4**

Enthalpy and Standard Deviation **5**

UMAP of SOAP descriptors **6**

Grain Boundary Analysis **7**

Machine learning interatomic potential training

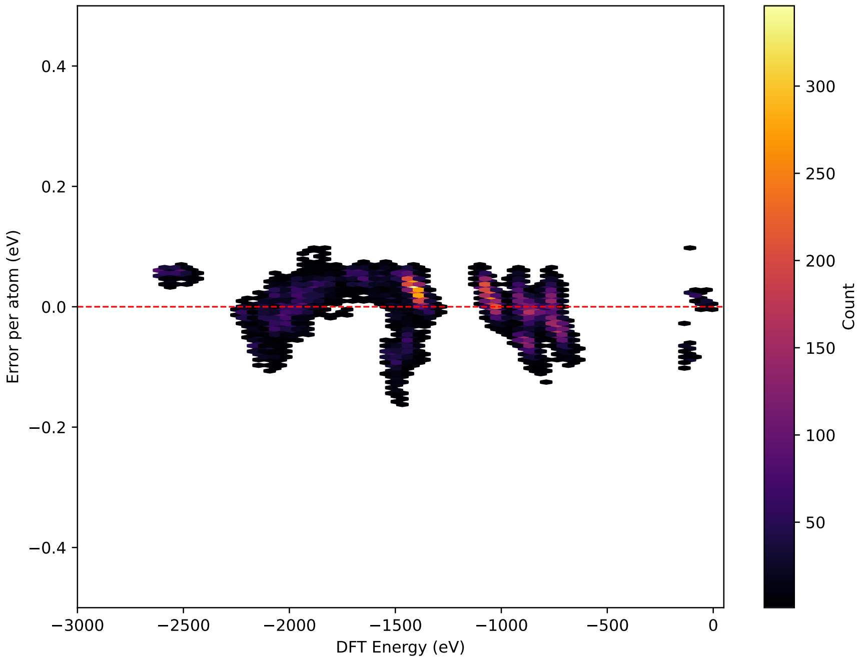

Figure S1.1: MLIP energy error for the dataset against the DFT ground truth

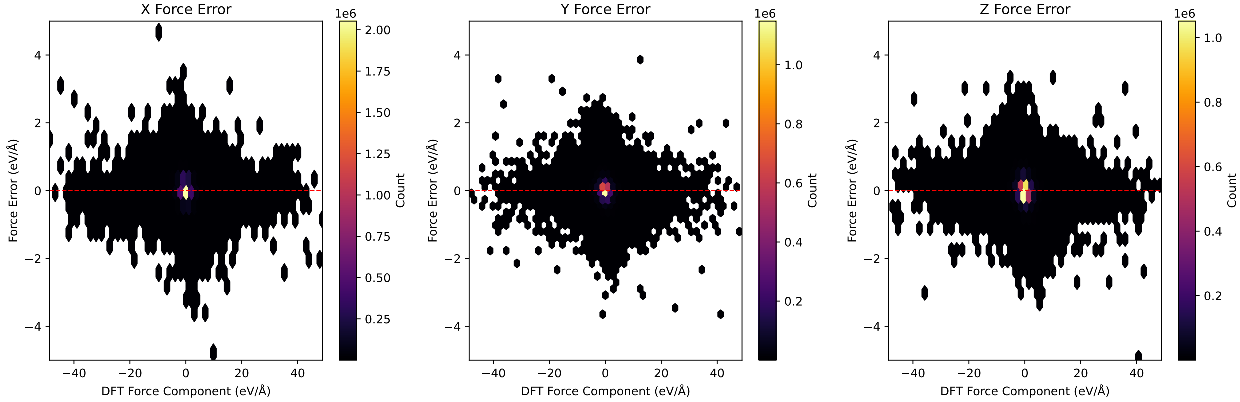

Figure S1.2: MLIP force component error for the dataset against the DFT ground truth

Surface and grain boundary energies

Table S2.1: Calculated surface energies of the DFT ground truth and the MLIP

Surface	DFT (eV/Å^2)	ML (eV/Å^2)
100	0.110	0.114
110	0.237	0.203
111	0.244	0.210

Table S2.2: Calculated grain boundary energies of the DFT ground truth and the MLIP. Idealized grain boundaries are formed from the stacking of surfaces, annealing with GCMC using the MLIP, and finally geometry optimization of these structures with the respective methods.

GB	DFT (eV/Å^2)	ML (eV/Å^2)
100-110	0.136	0.196
110-111	0.093	0.133
110-111	0.103	0.137

Enthalpy and Standard Deviation

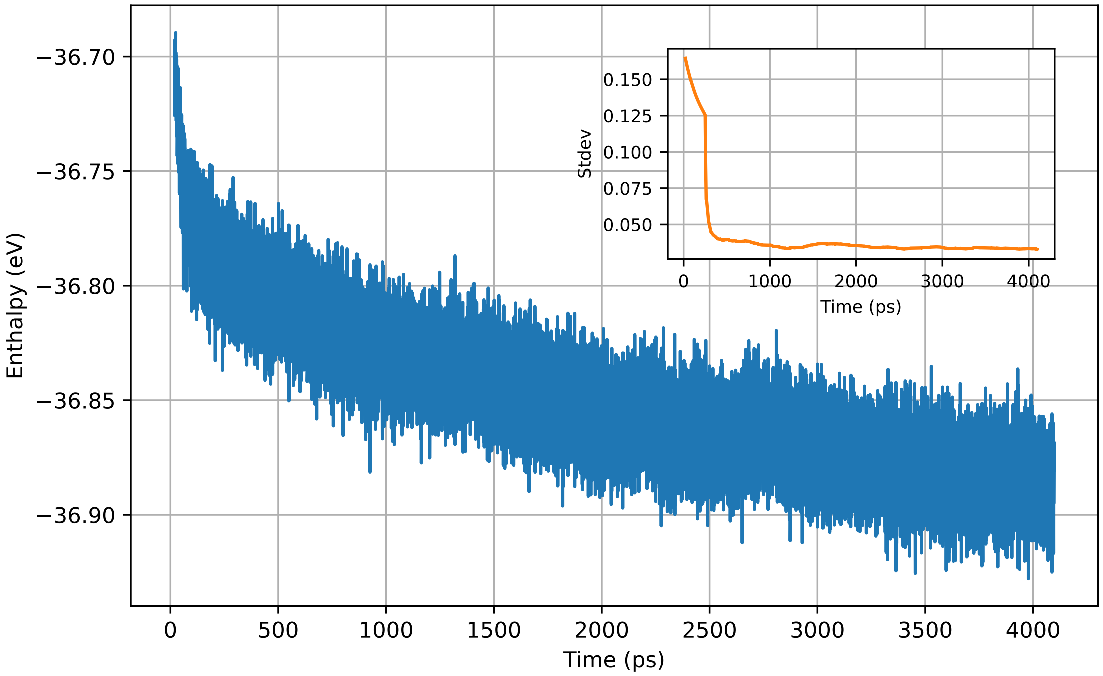

Figure S3: Enthalpy change of densification. The standard deviation of the enthalpy is also shown to verify convergence.

UMAP of SOAP descriptors

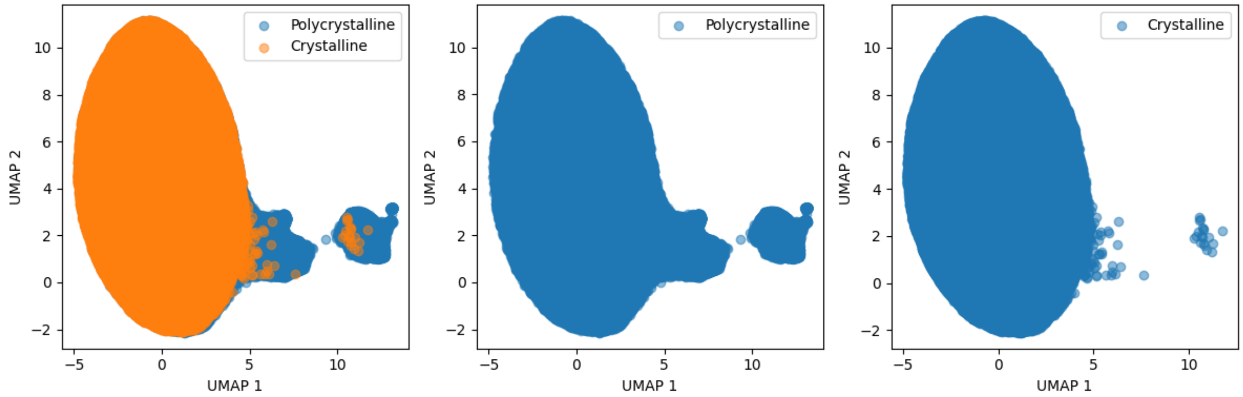

Figure S4: UMAP plot of SOAP descriptors reduced to two components. The marked polycrystalline points are for the pseudo-pellet oxygen atoms. The marked crystalline points are from oxygen atoms in snapshots of a bulk crystalline trajectory.

Grain Boundary Analysis

Grain boundaries were identified from the Zr sublattice of the $BaZrO_3$ simulation using a local structural fingerprinting method. For each Zr atom, the six nearest oxygen neighbors were identified and their unit displacement vectors were used to build a fingerprint representing the local ZrO_6 octahedron. To evaluate how well each octahedron was preserved, the six oxygen neighbors were grouped into three opposing pairs and the mean absolute cosine of the corresponding O–Zr–O angles was calculated. Values close to 1 indicate a well-ordered octahedron, while lower values indicate increasing local disorder. Crystalline Zr atoms were then grouped into individual grains using a breadth-first search (BFS) clustering procedure on the Zr sublattice graph. Two crystalline Zr atoms were considered connected if each was among the other's eight nearest Zr neighbors and if their octahedral fingerprints had a cosine similarity greater than or equal to the chosen threshold (0.80). Each connected component in this graph was taken to represent a grain. Atoms falling below the octahedral quality threshold, and were labeled assigned GB atoms. Misorientation between neighboring grains was calculated as the disorientation angle, defined as the minimum misorientation across all symmetry-equivalent representations under cubic symmetry. In addition, each boundary was labeled as tilt, twist, or mixed based on the relationship between the symmetry-reduced rotation axis and the boundary plane normal, which was approximated by the vector connecting the centroids of the two grains. The twist fraction was defined as the absolute dot product between the rotation axis and the boundary normal. Boundaries with twist fraction < 0.3 were classified as tilt, those > 0.7 as twist, and intermediate values as mixed.

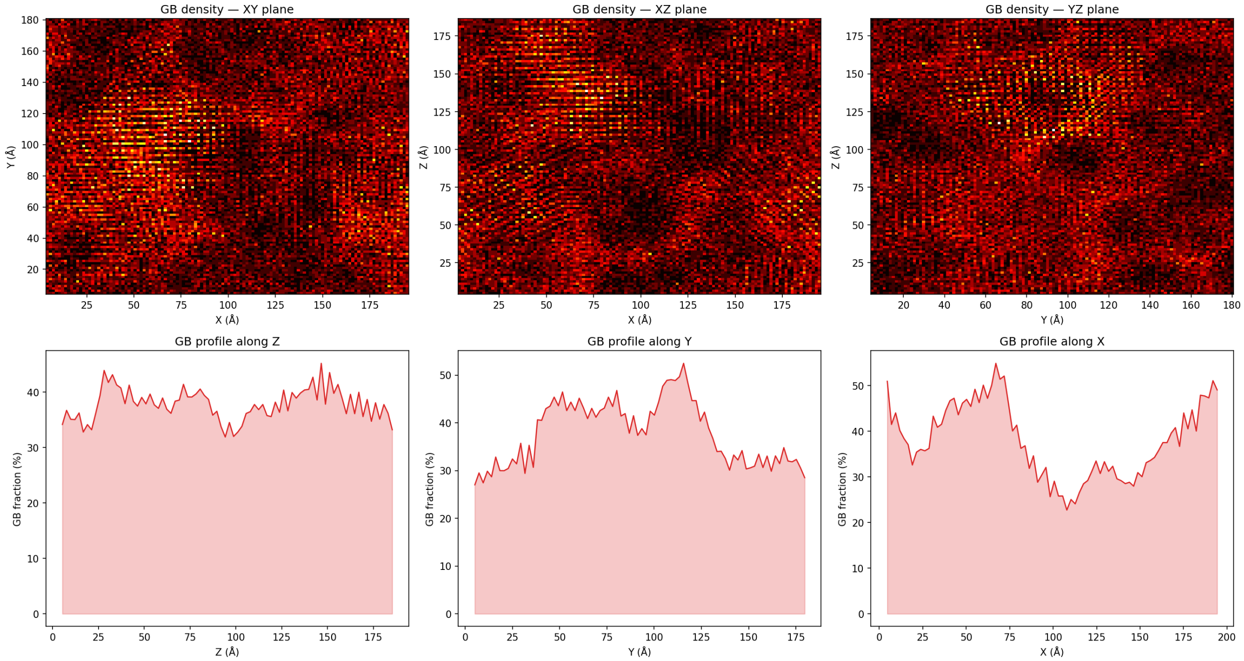

Figure S5.1: Spatial distribution of grain boundary atoms in the $BaZrO_3$ simulation cell. Top row: 2D density maps of GB atom positions projected onto the XY, XZ, and YZ planes. Bottom row: 1D GB fraction profiles along each axis. The heatmap shows the number of GB atoms per bin, ranging from low (black) to high GB density (red).

Table S5.4: Diffusion coefficients for the generated idealized grain boundaries, calculated at 1300 K.

GB	Diffusion coefficient (cm^2/s)
100-110	2.206 E-05
110-111	1.531 E-05
110-111	4.668 E-06

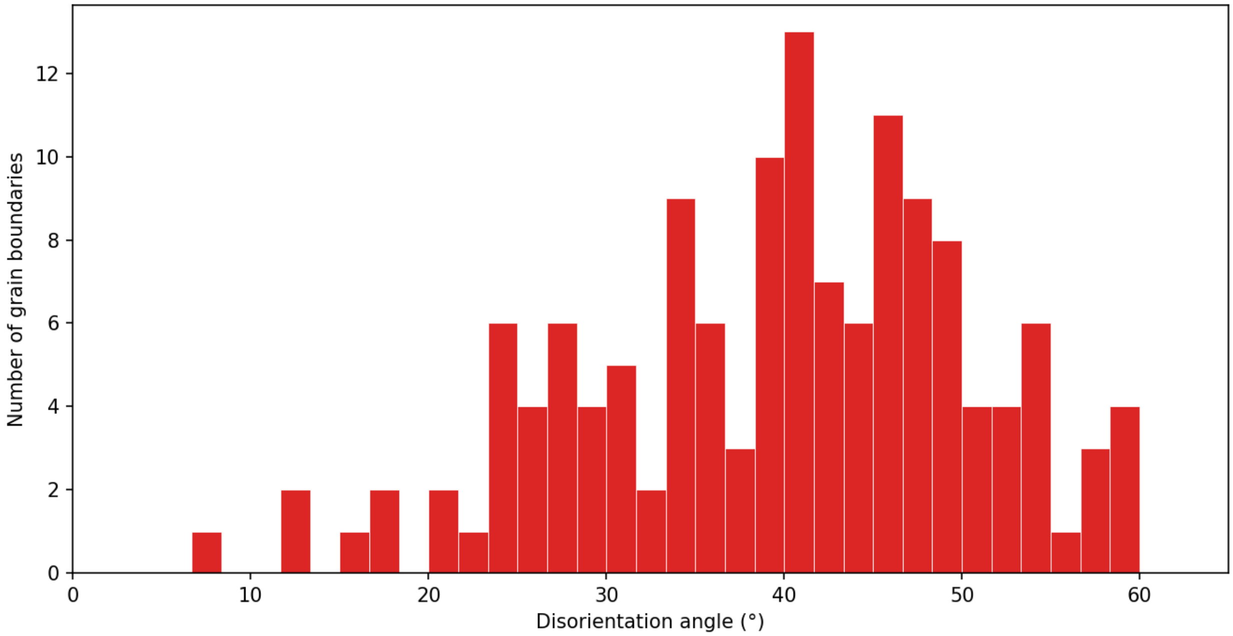

Figure S5.2: Distribution of grain boundary disorientation angle of the formed grain boundaries in $BaZrO_3$.

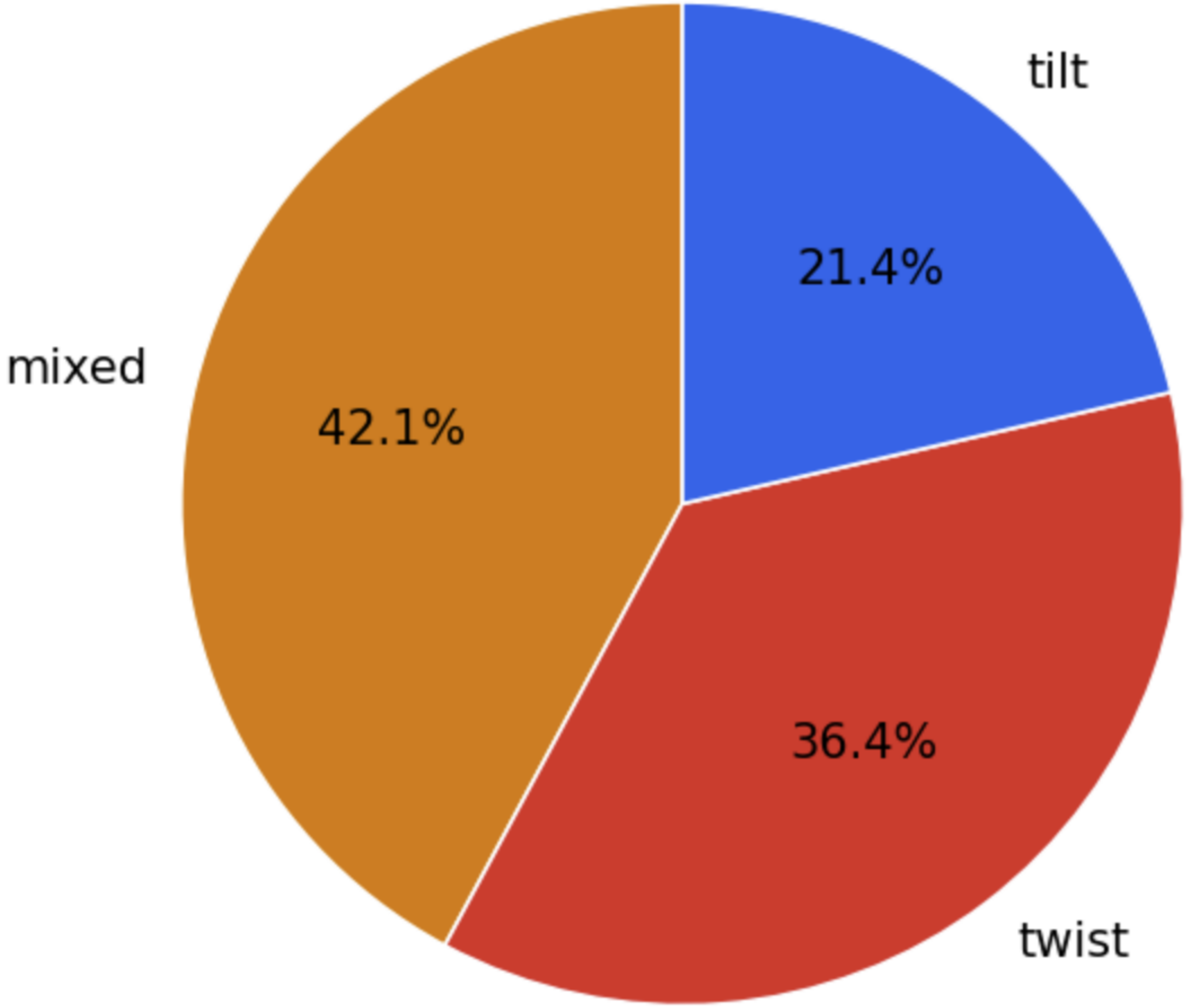

Figure S5.3: Classification of the grain boundaries into tilt, twist, or of mixed character, based on the relationship between the symmetry-reduced disorientation axis and the boundary plane normal.
